# Metabolic and Inflammatory Biomarkers Predicting Sarcopenic Obesity and Cardiometabolic Risk in Arab Women: A Cross-Sectional Study

**DOI:** 10.3390/ijms26125699

**Published:** 2025-06-13

**Authors:** Gregory Livshits, Nader Tarabeih, Alexander Kalinkovich, Adel Shalata, Shai Ashkenazi

**Affiliations:** 1 Department of Morphological Sciences, Adelson School of Medicine, Ariel University, Ariel 4070000, Israel; nadertar@gmail.com (N.T.); shaias@ariel.ac.il (S.A.); 2 Department of Anatomy and Anthropology, Gray Faculty of Medical and Health Sciences, Tel-Aviv University, Tel-Aviv 6905126, Israel; alexander.kalinkovich@gmail.com; 3 Department of Nursing, The Max Stern Yezreel Valley College, Emek Yezreel 1930600, Israel; 4 The Simon Winter Institute for Human Genetics, Bnai Zion Medical Center, The Ruth and Bruce Rappaport Faculty of Medicine, Technion, Haifa 3200003, Israel; adel.shalata@gmail.com

**Keywords:** sarcopenia, obesity, inflammation, hypertension, diabetes, hyperlipidemia

## Abstract

The sarcopenic obesity-related phenotype (SOP) is defined by the coexistence of sarcopenia and obesity, leading to heightened disability, morbidity, and mortality. Its multifactorial pathogenesis involves chronic inflammation and metabolic alterations. In this cross-sectional study, 562 women were classified into four groups: control, sarcopenic, obese, and SOP. Body composition measurements, including fat mass, skeletal muscle mass, and extracellular water (ECW), were assessed using the bioimpedance method. Several inflammatory biomarkers were measured in plasma samples by ELISA. Discriminant function analysis identified age, ECW, chemerin, the systemic immune-inflammation index (SII), and the ratio of total cholesterol to high-density lipoprotein cholesterol (TC/HDL-C) as significant discriminators among groups, clearly distinguishing SOP from control. Multivariable logistic regression analysis revealed that these variables were independently associated with SOP status (SOP vs. control), regardless of age, with odds ratios (ORs) ranging from 1.87 (95% confidence interval [CI]: 1.23–2.85) for SII to 7.77 (95% CI: 3.67–16.44) for ECW. A generalized estimating equation (GEE) analysis further demonstrated that SOP significantly increased the odds (OR: 3.04; 95% CI: 1.39–6.67) of multimorbidity (hypertension (HTN) + hyperlipidemia (HLD) + type 2 diabetes (D2T)). These findings suggest SOP is a clinically relevant phenotype linked to cardiometabolic comorbidities and systemic inflammation. Identifying SOP using accessible body composition and biomarker assessments may support early risk stratification and guide personalized preventive strategies in clinical care.

## 1. Introduction

Sarcopenic obesity (SO) is a clinical condition typically defined by the coexistence of sarcopenia, characterized by low skeletal muscle mass (SMM) and diminished muscle function, and obesity, which is marked by excess fat mass (FM) or an elevated waist circumference (WC) measurement [1]. Unlike sarcopenia (SP) or obesity (OB) alone, SO is associated with a synergistic aggravation of muscle loss and fat mass accumulation, driven by aging, physical inactivity, and shared pathogenic factors, such as chronic inflammation, insulin resistance, diabetes, etc. Consequently, SO significantly increases the risk of metabolic disease, functional impairment, and mortality relative to either condition alone [2,3,4]. Moreover, the prevalence of SO varies considerably among different ethnic groups [5,6], highlighting the need for population-specific investigations.

Globally, the prevalence of SO is increasing in parallel with population aging and rising obesity rates [7,8]. Current estimates suggest that 10–20% of older adults may be affected by this dual condition, depending on the population studied and the criteria applied [7]. In the Middle Eastern populations, including Israeli Arabs, the burden of obesity and cardiometabolic diseases is well documented [9,10,11,12]. However, data on sarcopenia and SO in these groups remain limited.

Despite significant efforts, the molecular mechanisms underlying SO remain unclear. Recently, we proposed that the interplay between adipose tissue and SMM inflammation is a major driver of SO pathogenesis [13]. Supporting this idea, in obesity, adipocytes undergo hypertrophy and activation, leading to increased production of adipokines, with pro-inflammatory adipokines such as leptin and adipsin predominating over anti-inflammatory adipokines, such as adiponectin and vaspin [14,15]. Another interesting factor in this connection is chemerin, a unique adipokine, a multifunctional protein involved in adipogenesis, as well as in lipid and glucose metabolism [16,17], that can exhibit both pro-inflammatory and anti-inflammatory effects, depending on the disease and physiological context [18]. However, its role in SO remains unclear.

Enhanced adipogenesis in SO leads to the recruitment and activation of immune cells that primarily produce pro-inflammatory mediators such as follistatin-like-1 (FSTL1) [19] and monocyte chemoattractant protein 1 (MCP1) [20], but also secrete anti-inflammatory factors such as follistatin [21] and fibroblast growth factor-21 (FGF-21) [22], as well as molecules with dual pro- and anti-inflammatory activities, such as growth and differentiation factor-15 (GDF-15) [23].

In addition, adipose tissue in obesity is characterized by excessive lipid production, leading to its ectopic accumulation in skeletal muscle, which creates a lipotoxic environment and promotes insulin resistance. This is accompanied by enhanced secretion of certain pro-inflammatory myokines, such as activin A [24], which can induce muscle dysfunction in an autocrine/paracrine manner [25,26]. Furthermore, these myokines possess endocrine functions exacerbating adipose tissue inflammation [27], creating a self-perpetuating pro-inflammatory cycle that sustains and worsens the progression of SO [13]. The ongoing low-grade chronic inflammation in SO can be characterized by elevated blood levels of a variety of inflammatory biomarkers [28,29,30,31,32].

Recent studies indicate that SO is also associated with dyslipidemia, defined by elevated levels of plasma cholesterol, triglycerides (TG) and low-density lipoprotein cholesterol (LDL-C), along with reduced levels of HDL-C [33,34,35]. Additionally, accumulated findings suggest that changes in key body composition parameters, including FM and SMM, are integral to SO pathogenesis [8] and serve as key biomarkers for its diagnosis [1]. The SO-associated changes in body composition are accompanied by disturbed body fluid distribution [36,37], pivotal for the physiological status of an organism.

Given the shared inflammatory and metabolic basis of SO, T2D, and non-alcoholic fatty liver disease, lifestyle-based strategies such as the Mediterranean diet and lifestyle medicine have gained attention [38,39,40,41,42]. These interventions emphasize anti-inflammatory nutrition, physical activity, stress reduction, and behavioral support. They have demonstrated efficacy in managing insulin resistance, reducing hepatic steatosis, and improving body composition, thereby offering a potential framework for the prevention and management of SO.

Collectively, these findings suggest the existence of various biological factors and complex mechanisms associated with SO. Although none of these factors is unique to SO, their combined presence may serve as a useful diagnostic indicator. In the present study, we refer to an SOP, defined exclusively by body composition parameters, without direct assessment of muscle function, in line with recent reports [43,44]. Body composition was assessed using bioelectrical impedance analysis (BIA), a non-invasive method for estimating body compartments, including SMM-to-weight ratio (SMM/WT), FM-to-weight ratio (FM/WT), and ECW [45,46,47,48,49,50]. SOP was operationally defined by a low SMM/weight ratio (<30%) and abdominal obesity (WC ≥ 88 cm). Several derived indices were also evaluated, including SII and TC/HDL-C, and adipokines such as chemerin and leptin. These variables were analyzed for their potential utility in differentiating body composition phenotypes and predicting the presence of metabolic comorbidities.

The study had two primary aims. The first was to identify biological risk factors associated with SOP. The second was to evaluate the association between SOP and metabolic comorbidities, including HTN, HLD, and T2D.

## 2. Results

### 2.1. Group Characteristics and Trends

A total of 562 females were classified into four phenotypic groups based on body composition parameters. Comparisons between groups and basic descriptive statistics for the study variables are summarized in Table 1. The groups were ordered based on their mean age; the trends (if any) were tested after adjusting for age, and *p*-values were corrected for multiple testing implementing the Bonferroni method.

Compared to healthy individuals, participants in the SP, OB, and SOP groups were older and exhibited significantly higher obesity-related measures, including body mass index (BMI) and FM/WT (all *p* < 0.001 after adjustment for age). ECW levels were also significantly elevated in all three non-control groups, indicating a systemic fluid imbalance associated with altered body composition.

Plasma levels of chemerin, GDF-15, and SII index were significantly elevated across groups with a clear age-independent trend. Adiponectin levels consistently decreased (*p*-trend = 0.006, not significant after correction for multiple testing), while adipsin levels demonstrated a similar but non-significant trend. Chemerin, in particular, demonstrated a progressive increase from the control group to SOP, with a highly significant trend (*p* = 0.000006), suggesting a dose–response relationship with SOP severity. Fasting glucose, TG, and TC levels were elevated across the groups (*p* < 0.01), with a significant trend even after adjustment for age, but not after the correction of multiple testing. The ratio of TC/HDL-C, however, showed a clear progressive increase, which remained significant after adjustment for age (*p* = 1.30 × 10^8^) and Bonferroni correction.

Regarding comorbidities, the prevalence of comorbid conditions differed significantly across the four phenotypic groups (Table 1), with the SOP group exhibiting the highest overall burden, ranging from 26.80% (T2D) to 46.31% (HLD). In contrast, the control group showed substantially lower prevalence rates (from 1.47% to 4.41%, respectively). The differences were statistically significant (χ^2^ test, all *p* < 0.001), highlighting the strong clustering of metabolic comorbidities within the SOP phenotype.

### 2.2. Discriminant Function Analysis (DFA)

**Figure 1:**
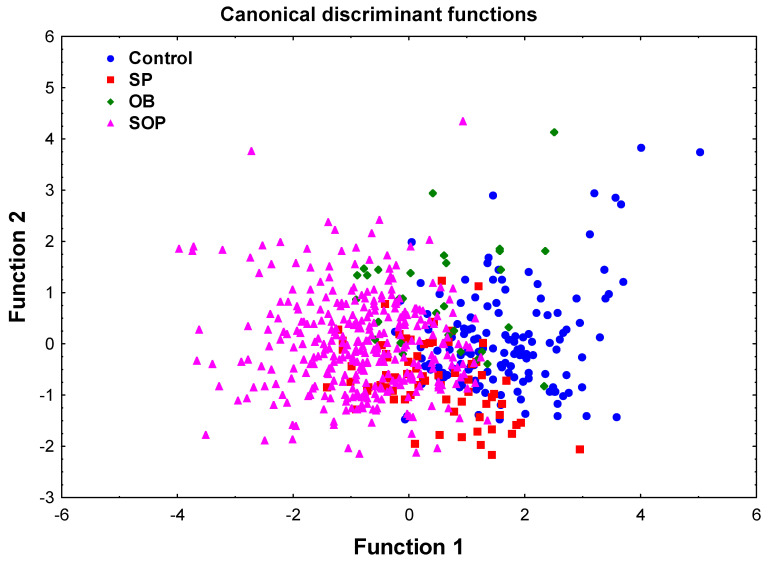
Scatterplot of canonical discriminant functions (function 1 vs. function 2). The plot of discriminant functions 1 and 2 established by the canonical discriminant analysis (CDA) derived from the analysis of four groups: healthy controls, sarcopenic-only (SP), obese-only (OB), and sarcopenic obesity-related phenotype (SOP). Each group is represented using different symbols and colors to show the spatial distribution and separation across the two discriminant roots. Classification accuracy was highest in the SOP group (93.3%) and controls (78.7%), while the SP (50.0%) and OB (0.0%) groups showed poorer separation. Overall, the model correctly classified 74.9% of the participants, indicating strong discriminative power among the groups. These results underscore the ability of the discriminant model to effectively separate individuals with SOP from other phenotypes while highlighting partial overlap among the remaining groups.

To assess the covariates’ ability to differentiate between the four study groups, a stepwise-forward DFA was conducted using significant covariates detected in univariate analyses. The final model (Table 2) achieved a Wilks’ Lambda of 0.41 (*p* < 0.00001), indicating a strong discriminative power of the four retained in the discrimination covariates, ECW (*p* = 9.13 × 10^11^), chemerin (*p* = 0.001), SII (*p* = 0.008), and TC/HDL-C ratio (*p* = 8.72 × 10^9^), independent of age. Figure 1 displays this result graphically using the canonical discriminant function plot, showing a clear distinction of the SOP group from the control group. The classification results are impressive, with an overall model classification of 74.9% of the participants; however, these results were not clearcut. The low classification accuracy for the SP and especially for the OB individuals is likely due to their intermediate characteristics and small sample size. Therefore, the next stage of analysis is focused solely on comparing the SOP and control groups.

### 2.3. Comparison Between the Control and SOP Groups

**Figure 2:**
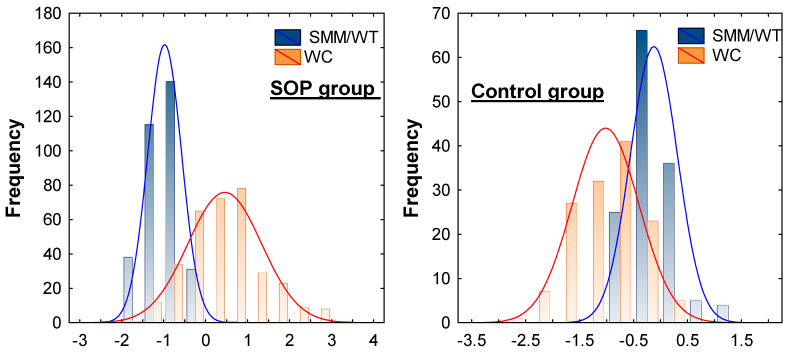
Distribution of standardized skeletal muscle mass/weight ratio (SMM/WT) and waist circumference (WC) scores in the sarcopenic obesity-related phenotype (SOP) group vs. control, adjusted for age prior to comparison. The difference in the pattern of both measurements’ distribution is clearly seen. The contrasting distributions visually illustrate the defining characteristics of SOP compared to healthy individuals.

Figure 2 presents the distribution of the standardized SMM/WT and WC scores in the SOP group vs. control group, adjusted for age. Participants with SOP were significantly older than healthy controls (49.47 ± 0.65 vs. 30.26 ± 0.73 years, *p* = 1.63 × 10^8^) and exhibited markedly higher BMI (32.36 ± 0.26 vs. 21.88 ± 0.20 kg/m^2^). The SOP group also showed significantly elevated FM/WT and ECW levels (all *p* < 0.001). Among the biochemical markers, chemerin and leptin levels were significantly elevated in the SOP group as compared to the control group, reinforcing their role in fat–muscle crosstalk and adiposity-linked inflammation. Conversely, adiponectin levels were significantly reduced in the SOP group (*p* = 0.0002). Markers of systemic inflammation, such as leukocyte count and SII, were also significantly elevated in the SOP group compared to the control group, even after adjustment for age (*p* < 0.001). Participants with SOP exhibited significantly higher levels of fasting plasma glucose, TG, and TC, along with lower HDL-C levels, compared to the control group (*p* < 0.01). These markers also demonstrated notable trends across the groups. When the TC/HDL-C (mg/dL) ratio was compared between the SOP and control groups, a highly significant difference was observed after adjusting for age (5.75 × 10^11^).

### 2.4. Multivariable Logistic Regression Analysis for SOP Risk

We first conducted multiple logistic regression analyses to evaluate the extent of the independent associations of the selected covariates with the SOP status (Table 3). The results of this analysis suggested that ECW and chemerin levels, SII, and TC/HDL-C ratio were significant and independent of each other, and age was associated with SOP. The corresponding OR ranged between 1.87 (95%CI 1.23–2.85) for SII and 7.77 (95%CI 3.67–16.44) for ECW. Notably, all the covariates demonstrated a strong positive association with SOP.

### 2.5. Comorbidity Associations

**Figure 3:**
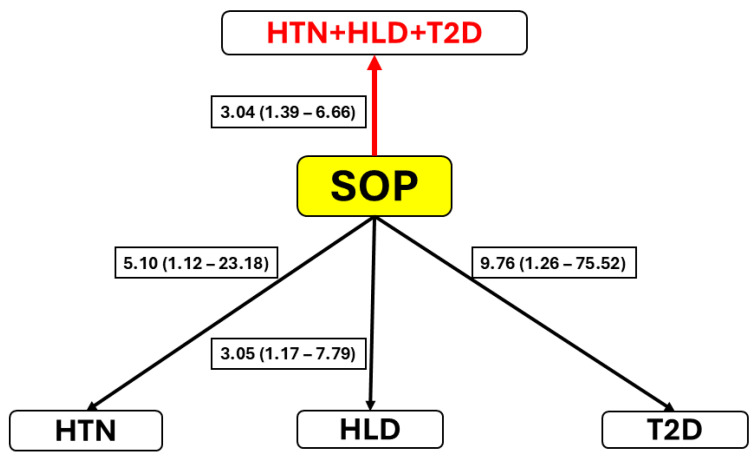
Associations between SOP and cardiometabolic comorbidities. The diagram illustrates the associations between SOP and four comorbid outcomes: hypertension (HTN), hyperlipidemia (HLD), type 2 diabetes (T2D), and a combined multimorbidity outcome (HTN + HLD + T2D). The red arrow reflects a significant relationship estimated using a generalized estimating equation (GEE) model, capturing the influence of SOP (simultaneously adjusted for age) as independent variable on the likelihood of having any of three conditions. Black arrows represent significant associations between SOP (adjusted for age) and each of the comorbidity conditions estimated in the separate binary logistic regression analyses. Odds ratios (ORs) with 95% confidence intervals (CIs) displayed on arrows.

Table 1 clearly showed higher prevalence of the three comorbidities in the SOP group in comparison to the other three groups, but in particular to controls. It was therefore important to evaluate the extent to which SOP is associated with each of the comorbidity categories, independently of age. The results of three independent multiple logistic regression analyses are summarized in Figure 3. The figure displays the age-adjusted ORs, which were highly statistically significant in all instances. However, the occurrence of comorbid conditions was not independent. The chi-square tests showed their significant (*p* < 0.001) pairwise associations, with Phi coefficients ranging from 0.457 to 0.576 (Table 4). To account for this non-independence of comorbidities, we implemented multivariate logistic regression for testing the association of SOP with the combined multimorbidity.

The GEE model was employed, and results are shown in Figure 3. After restructuring the dataset into a long format to accommodate multiple outcomes per participant, SOP was found to have a significantly higher OR of 3.04 (95% CI 1.39–6.66, *p* = 0.005) being in one of the disease groups compared to the control group. Higher age values were also associated with increased odds across the outcomes, with an OR of 5.03 (95% CI 3.85–6.58, *p* < 0.001). In summary, these results provide evidence that the presence of SOP, independent of increasing age, is a significant risk factor contributing to the likelihood of developing HTN, HLD, and T2D relative to the control.

## 3. Discussion

To study the SOP in Arab women, we applied a complex approach, simultaneously detecting body composition, inflammation, and metabolic characteristics, along with testing the hypothesis that SOP is associated with the elevated prevalence of the metabolic comorbidities. Our paper reports several novel findings: (1) SOP is significantly and independently associated with plasma levels of adipokines, specifically chemerin, inflammation index, SII, lipid profile (TC/HDL-C ratio), and body composition characteristics (ECW); (2) SOP, in turn, is a significant risk factor contributing to the likelihood of being associated with HTN, HLD, and T2D.

This study, for the first time, reports a significant association between chemerin and SOP. Chemerin is a pleotropic adipokine involved in inflammation, adipogenesis, angiogenesis, and energy metabolism [16,17]. However, regarding inflammation, its role is controversial, as it seems to play both pro-inflammatory and anti-inflammatory roles, depending on the status of the disease [18,51,52]. An increase in circulating levels of chemerin in obesity has been shown to lead to a low-grade chronic inflammatory state in association with the development of cardiovascular disease [53,54,55,56]. It has also been shown to induce mitochondrial dysfunction in skeletal muscle [57] and inhibit insulin-sensitive glucose transport in muscular cells [58], presumably via activation of the pro-inflammatory NF-kB pathway [59]. A correlation between circulating chemerin levels and inflammation markers, such as high-sensitivity C-reactive protein, interleukin-6, and tumor necrosis factor α, has also been reported [60,61]. Interestingly, this association appears to be independent of body fat accumulation [62]. This suggests a possible role of chemerin in the modulation of the inflammatory process, as well as its contribution to inducing extensive inflammatory processes in various inflammatory disorders. However, chemerin also appears to have anti-inflammatory properties, by acting on its receptors expressed by non-leukocytic cells, such as endothelial cells, liver and skeletal muscle [63]. Therefore, a clear association of elevated chemerin levels with inflammation-associated SOP in our study demands further elucidation.

An additional novel finding in our study is the strong association of SOP with ECW levels, as demonstrated by a high OR value of 7.77 (95%CI 3.67–16.44) observed in the logistic regression analysis. ECW accounts for about 35% of the total body water. Changes in ECW osmolarity are accompanied by an outflow of water and, presumably, key biochemical factors from cells, leading to cell shrinkage, oxidative stress, protein alterations, mitochondrial and DNA damage, and cell cycle arrest, making cells susceptible to apoptosis [64]. ECW leakage has been associated with many chronic inflammatory disorders, including obesity [65] and arthritis [66]. Elevated ECW levels have also been shown to be independently associated with sarcopenia characteristics such as muscle strength, functional capacity, gait speed, and frailty [67,68,69]. Our data implicate elevated ECW levels in the development of SOP, although the underlying mechanisms remain unclear.

We also examined a range of inflammation-related markers, some of which, e.g., GDF-15 and leukocyte and neutrophil counts, showed a significant association with SOP in a univariate analysis. However, only SII remained significant after adjustment for age in multivariable logistic regression analysis. SII was developed to provide more comprehensive data on inflammation [70] and its elevated levels have been associated with worse prognoses for several medical conditions and higher mortality in patients with cardiovascular disease [71]. Recent cross-sectional studies have reported an association of elevated SII with sarcopenia [29], obesity [72], and SOP [28]. The latter study suggests that systemic inflammation affects SOP by reducing insulin sensitivity, which in turn reduces muscle protein metabolism and adipose tissue function, leading to decreased and increased fat mass, respectively.

Impaired lipid profile has been repeatedly observed in SOP patients [73,74]. In this regard, significant and independent association of SOP with TC/HDL-C ratio, which is one of the strongest markers of cardiovascular risk [75], is of special interest. This ratio has been found to be significantly associated with obesity [76] and sarcopenia [77]; however, similar data on its association with SOP are extremely limited or not reported yet.

As mentioned in the Introduction, SOP is frequently associated with chronic comorbidities [29]. In the present study, we observed a markedly increased prevalence of each of the three metabolic conditions examined—HTN, HLD and T2D—in the SOP group compared to all other groups, and particularly in comparison to the control group. Univariate and multivariate logistic regression analyses corroborated these observations (Figure 3). Regardless of the design of the analysis, whether comorbidities were evaluated independently or as a diagnosis group, the associations with SOP were consistently and statistically significant. These findings strongly suggest the involvement of metabolic dysregulation, associated with the development of SOP, in the pathogenesis of chronic metabolic disease. This is not unexpected, given that key risk factors for SOP, in particular obesity, inflammation, and dysregulated lipid metabolism, are also implicated in the etiology of the comorbidities assessed in this study. Supporting this interpretation, numerous cross-sectional studies have demonstrated associations between SOP and elevated risk for HTN, dyslipidemia, T2D, and metabolic syndrome (reviewed in [8,78,79,80,81]). However, findings from a limited number of prospective, longitudinal studies have been inconclusive regarding the link between SOP and cardiovascular outcomes [82,83]. Taken together, our findings suggest that SOP may serve as a composite risk marker for metabolic diseases.

This study has several limitations that should be acknowledged. First, its cross-sectional design precludes the establishment of temporal or causal relationships between SOP, inflammatory and metabolic biomarkers, body composition parameters, and comorbidities. Prospective, longitudinal studies are needed to clarify the directionality of these associations and to evaluate their prognostic significance. Second, the study population consisted exclusively of Arab women from a single geographic region, which may limit the generalizability of the findings to other ethnic, cultural, or geographic groups. Third, there is potential for residual conditions, such as physical activity levels, dietary habits, smoking status, stress, and quality of life, which were not assessed in this study. Fourth, although established body composition thresholds were used to define SOP, muscle function (e.g., handgrip strength) was not directly assessed, limiting the applicability of the findings to clinical definitions of SO. Fifth, the present analysis was based on a sample of 562 women. While the sample size exceeds commonly accepted thresholds for both univariable and multivariable modeling, future studies should employ prospectively designed cohorts to validate and expand these findings.

In conclusion, this study reports several novel findings with prognostic potential for SOP. It demonstrates that SOP is a highly prevalent and metabolically harmful condition in Arab women, marked by distinct alterations in body composition and inflammatory markers. In turn, ECW and chemerin levels, the SII, and the TC/HDL-C ratio emerged as reliable predictors of SOP, offering potential targets for future diagnostic and therapeutic strategies. These findings have important clinical implications, suggesting that a comprehensive assessment of body composition and inflammatory markers should be integrated into routine evaluations to identify individuals at increased risk and to inform personalized management strategies.

Perspective for Clinical Practice: The findings of the present study, in conjunction with existing evidence, underscore important implications for clinical practice. The identification of SOP through accessible, non-invasive measures such as BIA and basic anthropometric indices (e.g., waist circumference) may facilitate early detection of individuals at heightened cardiometabolic risk. This approach is particularly feasible in primary care and community health settings [84,85]. Integrating SOP screening into routine clinical assessments could inform targeted prevention strategies aimed at mitigating the risk of HTN, HLD, and T2D [35,86,87]. Furthermore, the incorporation of biomarkers including chemerin, SII, and ECW, each of which demonstrated strong discriminatory capacity in our analysis, may enhance understanding of the underlying pathophysiology and support the development of personalized monitoring frameworks [69,88,89].

From a broader perspective, these findings underscore the need for integrated clinical approaches that combine metabolic, inflammatory, and body composition data to inform early intervention. SOP assessment may serve as a tool to guide individualized lifestyle interventions, such as structured physical activity nutritional counseling (e.g., the Mediterranean diet) and other evidence-based lifestyle medicine strategies aimed at mitigating systemic inflammation and improving muscle–fat balance [90,91,92]. Future research should explore the feasibility, cost-effectiveness, and clinical utility of implementing SOP-based screening and intervention strategies in real-world healthcare settings.

## 4. Materials and Methods

### 4.1. Study Sample and Ethics

This cross-sectional study was conducted and reported in accordance with STROBE (Strengthening the Reporting of Observational Studies in Epidemiology) guidelines for observational studies, as detailed in Supplementary File S1 [93].

This community-based study included 562 women (mean age: 43.66 years; range: 18–70 years) recruited between January 2016 and January 2024 from outpatient clinics in Sakhnin, Israel. These clinics provide general healthcare services, including primary care and family medicine. The study sample represents an ethnically and culturally homogeneous population of Israeli Arabs. Participants were recruited consecutively from a larger cohort originally established to investigate low back pain and its associated clinical and biological features. Women attending the clinics during the recruitment period were invited to participate in the research project, regardless of any health or physical characteristics. All participants provided informed consent and allowed access to their medical records or completed a comprehensive medical history.

The inclusion criteria included randomly collected women with respect to body composition. Exclusion criteria included pregnancy, age under 18 years, and the presence of systemic inflammatory or autoimmune conditions.

All participants underwent clinical screening by trained nurses, including blood sampling and assessment of comorbidities. Blood samples were used to assay plasma concentrations of biochemical factors relevant to the present study.

This study was approved by the IRB-Helsinki Committee of Meir Medical Center (Approval No. 042/2013K) and the Ethics Committee of Tel Aviv University. Written informed consent was obtained from all participants.

SOP was defined by the coexistence of SP, characterized by low SMM, and OB, based on body composition criteria derived from BIA, specifically low SMM/WT < 30% and abdominal obesity (WC ≥ 88 cm) [43,44]. Individuals meeting both criteria were classified as having SOP. The study sample was categorized into four groups: 70 individuals with SP (but not obese, WC < 80 cm), 30 individuals with OB (but not SP, SMM/WT > 0.36), 326 individuals with SOP, and 136 healthy controls, free of SP, OB, and SOP.

### 4.2. Demographic, Anthropometric, and Body Composition Assessment

These data collections have been recently described in detail [49,94,95]. Briefly, anthropometric measurements consisted of height (cm), weight (kg), BMI in kg/m^2^, and WC (cm). Body composition was assessed using BIA with the BIA101 device (Akern Bioresearch, Florence, Italy), a safe, reliable, accurate, and inexpensive method, as described [95,96,97], providing evaluation of FM and SMM in kg, and ECW in liters. FM/WT and SMM/WT ratios were calculated to adjust for body weight.

### 4.3. Measurement of Plasma Levels of Inflammatory Biomarkers

Venous blood samples were collected from all study individuals after an overnight fast. Within 1 h of collection, they were centrifuged for 15 min at 1800× *g* at 4 °C. Plasma fractions were separated and stored in aliquots at −80 °C. Levels of biomarkers were determined by ELISA using the DuoSet kits (R&D Systems, Minneapolis, MN, USA) according to the manufacturer’s protocols. The detection limits were as follows: 7.8 pg/mL for GDF-15, 46.9 pg/mL for follistatin, 31.2 ng/mL for chemerin, 31.2 pg/mL for leptin, 62.5 µg/mL for adiponectin, 375 µg/mL for adipsin, 49.6 pg/mL for vaspin, 0.3 ng/mL for FSTL1, 31.2 ng/mL for FGF-21, 15.6 pg/mL for MCP1, and 125.0 pg/mL for Activin A. The intra- and inter-assay coefficients of variation were between 2.3 and 8.6%. Before statistical analysis, the original measurements of the biomarkers deviating from the normal distribution assumptions were log-transformed.

### 4.4. Blood Glucose Levels, Lipid Profile, and Blood Count

Fasting blood samples were obtained for complete blood counts, glucose, and blood lipids measurement, including TG, TC, HDL-C, and LDL-C. The blood count was used for the evaluation of inflammatory indices.

### 4.5. Inflammatory Indices

The study examined several inflammatory indices, based on the counts of total leukocytes, lymphocytes, monocytes, neutrophils, and platelets. While platelets are well-known blood clotting factors, emerging evidence highlights their significant contribution to immune regulation and inflammatory processes [98]. The following inflammation-related indexes were calculated using the above counts: (1) SII = platelets x neutrophils/lymphocytes ratio [99]; (2) the systemic inflammation response index (SIRI = neutrophils x monocytes)/lymphocytes) [100]; (3) monocyte-to-high-density-lipoprotein ratio (MHR) obtained by dividing monocyte count (10^3^ cells/μL) by HDL-C levels (mg/dL) [101,102].

### 4.6. Assessment of SOP-Related Comorbidities

The data in our possession allowed us to consider the following comorbidity categories: HTN, HLD, T2D. The clinical assessment of these conditions was adopted from our previously published studies [49,103].

### 4.7. Study Design and Statistical Analysis

The study design included two major stages: the examination of four body composition groups and the comparison of the SOP vs. the control group. The first stage included testing the following hypothesis and implementing the corresponding methods of analysis:

1. A comparison was conducted of all the potential covariates between the four study groups, implementing the ANCOVA method for testing the general trend and to compare SOP and control groups. The frequency of the comorbidities in the study groups was compared by the χ^2^ test. The association of the variable with SOP was considered significant after a multiple-comparison correction using the Bonferroni test.
2. Discrimination analysis. The covariates that survived multiple testing correction using Bonferroni method were next used to simultaneously evaluate the ability of selected variables to differentiate among groups. A discriminant function analysis (DFA) was performed using a stepwise linear discrimination procedure. The extent of the variable discrimination between the groups was evaluated by Wilks’ Lambda (0 value = perfect discrimination) and associated chi-square statistics. A scatterplot of canonical scores was used to visualize the separation between groups.
3. To further clarify the results, in the second stage of the study, we compared the SOP group with the control group, initially using multivariate logistic regression analysis. The independent variables were the same variables as those used in the discriminant analysis.
4. Next, we examined the association between the SOP status and the three comorbidities, HTN, HLD, and T2D. This analysis was, in turn, conducted in a few steps. First, the association of SOP, with simultaneous adjustment for age, and each of the comorbidities (independently of others) was tested implementing binary multiple logistic regression analysis. The major deficiency of this method is that it does not take into account the non-independence of the comorbidities. However, the study comorbidities were not independent of one another. The strength and direction of their pairwise relationships were examined by chi-square tests and the corresponding Phi coefficients.
5. Once non-independence was confirmed, we proceeded to a final step of the analysis: a multivariate logistic regression analysis of all three comorbidities, taking into account the extent of the associations between them, on SOP (and age). This analysis was conducted using a generalized estimating equation (GEE) approach to evaluate the association between the presence of SOP (1 = present, 0 = absent), standardized age (AGEz), and diagnosis group (any of the comorbidities, or their combination). The comparisons were made across HTN, HLD, and T2D, each of which was assessed as 1 (present) or 0 (absent). The data were restructured into a long format creating a new outcome binary variable representing presence (=1) of any of the three comorbidities (or their combination) vs. absence (=0) of all of them. An exchangeable working correlation structure was used in the GEE model to account for repeated measures within individuals. The results were expressed as ORs with 95% CIs, estimating the extent of the association of each of the predictors (SOP and age) with the comorbidity status.

All statistical analyses were performed using Statistica 64 (TIBCO Software Inc., Palo Alto, CA, USA; Version 14.0.1).

## Figures and Tables

**Table 1:** Basic descriptive statistics and comparison of assessed characteristics among study groups using ANCOVA with age as a covariate.

Study Group/ Variable	Control (n = 136)	SP (n = 70)	OB (n = 30)	SOP (n = 326)	P1	P2
Age (y)	30.26 ± 0.736	39.50 ± 1.273	40.37 ± 1.911	49.47 ± 0.654		
Anthropometric and Body Composition Variables						
BMI (kg/m^2^)	21.88 ± 0.196	25.59 ± 0.258	25.84 ± 0.516	32.36 ± 0.259	0.001 ^#^	0.001
FM/WT (kg)	0.27 ± 0.004	0.35 ± 0.003	0.30 ± 0.007	0.41 ± 0.002	0.001 ^#^	0.001
ECW (L)	14.84 ± 0.147	15.17 ± 0.215	16.41 ± 0.369	17.70 ± 0.136	1.61 × 10^14 #^	5.70 × 10^10^
Biochemical Factors						
Adiponectin (µg/mL)	4.82 ± 0.161	4.50 ± 0.21	4.33 ± 0.278	4.27 ± 0.094	0.005	0.0002
Adipsin (µg/mL)	0.09 ± 0.022	0.13 ± 0.028	0.20 ± 0.057	0.27 ± 0.016	NS	0.05
Activin A (pg/mL) *	7.05 ± 0.09	6.89 ± 0.13	6.65 ± 0.26	6.71 ± 0.06	NS	0.01
Follistatin (pg/mL) *	5.85 ± 0.082	5.87 ± 0.190	5.98 ± 0.190	6.25 ± 0.038	NS	NS
FSTL1 (ng/mL)	13.37 ± 0.38	13.66 ± 0.52	13.83 ± 0.64	13.77 ± 0.25	NS	NS
FGF-21 (ng/mL)	1.49 ± 0.04	1.45 ± 0.05	1.49 ± 0.09	1.49 ± 0.02	NS	NS
Chemerin (ng/mL)	73.28 ± 1.719	83.28 ± 2.800	85.66 ± 4.232	100.75 ± 1.532	0.000006 ^#^	0.00003
GDF-15 (pg/mL) *	8.20 ± 0.046	8.36 ± 0.067	8.38 ± 0.095	8.89 ± 0.040	0.01	NS
Leptin (ng/mL) *	2.64 ± 0.060	3.19 ± 0.066	2.94 ± 0.097	3.59 ± 0.032	0.001 ^#^	0.001
MCP1 (pg/mL) *	3.79 ± 0.05	3.77 ± 0.07	3.98 ± 0.08	3.89 ± 0.03	NS	NS
Vaspin (pg/mL)	5.92 ± 0.090	5.82 ± 0.128	6.26 ± 0.254	6.08 ± 0.069	NS	NS
Blood Cell Count and Inflammation Indexes						
Leukocytes (×10^9^/L)	6.19 ± 0.13	6.58 ± 0.23	6.59 ± 0.32	7.05 ± 0.11	0.00001 ^#^	0.000004
Neutrophils (×10^9^/L)	3.68 ± 0.117	4.79 ± 0.164	4.03 ± 0.261	4.17 ± 0.091	0.000002 ^#^	0.000001
Lymphocytes (×10^9^/L)	1.94 ± 0.04	2.06 ± 0.07	1.83 ± 0.11	2.17 ± 0.03	0.003	0.001
Monocytes (×10^9^/L)	0.41 ± 0.07	0.35 ± 0.01	0.33 ± 0.02	0.39 ± 0.02	NS	NS
Platelets (×10^9^/L)	244.00 ± 4.94	252.91 ± 8.72	263.80 ± 10.46	255.32 ± 3.84	NS	0.05
SII	460.85 ± 18.63	485.95 ± 25.39	537.99 ± 44.01	536.44 ± 19.28	0.000008 ^#^	0.000002
SIRI	0.69 ± 0.035	0.69 ± 0.048	0.78 ± 0.09	0.84 ± 0.056	NS	NS
MHR	6.60 ± 0.237	7.10 ± 0.410	6.92 ± 0.469	8.26 ± 0.394	NS	NS
Glucose and Blood Lipid Profile						
Fasting plasma glucose (mg/dL)	83.87 ± 0.738	88.10 ± 1.232	90.72 ± 2.330	102.75 ± 1.748	0.003	0.01
TC (mg/dL)	163.18 ± 2.33	178.68 ± 4.39	184.73 ± 6.68	185.04 ± 1.82	0.005	0.00004
TG (mg/dL)	80.31 ± 5.009	94.72 ± 5.509	98.61 ± 7.802	128.49 ± 3.901	0.0008 ^#^	0.0004
HDL-C (mg/dL)	54.04 ± 1.03	53.30 ± 1.58	49.95 ± 2.00	48.97 ± 0.63	0.00001 ^#^	0.000003
LDL-C (mg/dL)	96.22 ± 2.41	106.65 ± 3.80	114.80 ± 6.34	109.24 ± 1.70	0.008	0.0001
TC/HDL-C (mg/dL)	3.11 ± 0.065	3.49 ± 0.123	3.78 ± 0.160	3.89 ± 0.059	1.30 × 10^8 #^	5.75 × 10^11^
TG/HDL-C(mg/dL)	1.46 ± 0.078	1.92 ± 0.139	2.08 ± 0.202	2.90 ± 0.121	0.00001 ^#^	0.00001
Comorbidities, n (%)					χ^2^ test	χ^2^ test
HTN	2 (1.47)	6 (8.57)	2 (6.66)	125 (38.34)	<0.001	<0.001
T2D	4 (2.94)	2 (2.85)	3 (10)	87 (26.68)	<0.001	<0.001
HLD	6 (4.41)	10 (14.28)	7 (23.3)	151 (46.31)	<0.001	<0.001

Data are presented as mean ± standard error (SE) for continuous variables and as counts with percentages [n (%)] for categorical variables. The analysis compares four study groups: healthy controls, sarcopenic-only (SP), obese-only (OB), and sarcopenic obesity-related phenotype (SOP). Comparisons were performed using ANCOVA with age as a covariate. P1 presents *p*-values for comparisons across all groups, while P2 shows comparisons between SOP and control. The χ^2^ test was used to compare categorical variables (prevalence of comorbidities) between groups. Variables marked with **^#^** remained significant after Bonferroni correction for multiple testing. Biochemical markers were log-transformed to approximate normality and are marked with asterisks (*). Abbreviations: BMI, body mass index; ECW, extracellular water; FGF-21, fibroblast growth factor-21; FM/WT, fat mass/weight ratio; FSTL1, follistatin-like-1; GDF-15, growth and differentiation factor-15; HDL-C, high-density lipoprotein cholesterol; HLD, hyperlipidemia; HTN, hypertension; LDL-C, low-density lipoprotein cholesterol; MCP1, monocyte chemoattractant protein 1; MHR, monocyte-to-HDL-C ratio; N, sample size; NS, non-significant; SII, systemic immune-inflammation index (platelet × neutrophil)/lymphocyte ratio); SIRI, systemic inflammation response index (neutrophils × monocytes)/lymphocytes); T2D, type 2 diabetes; TC, total cholesterol; TC/HDL-C, total cholesterol-to-high-density-lipoprotein-cholesterol ratio; TG, triglycerides; TG/HDL, triglycerides-to-high-density-lipoprotein-cholesterol ratio; χ^2^, chi-square test.

**Table 2:** Assessment of individual variables’ contributions to discriminant analysis between study groups.

Covariates	Wilks’ Lambda	*p*-Value
Age	0.483	1.05 × 10^15^
ECW	0.427	9.13 × 10^11^
Chemerin	0.427	0.001
SII	0.431	0.008
TC/HDL-C	0.450	8.72 × 10^9^
	Overall model: Wilks’ Lambda = 0.41, *p* < 0.00001	

Wilks’ Lambda indicates the contribution of each variable in the discrimination between the study groups, with *p*-values from chi-square tests suggesting their high statistical significance, separately and in combination. Variables were standardized prior to analysis and tested using a stepwise-forward approach. The following predictors were evaluated initially: age, BMI, FM/WT, ECW, adiponectin, chemerin, leptin, TC/HDL-C, TG/HDL-C, neutrophils, and SII. Abbreviations: ECW, extracellular water; SII, systemic immune-inflammation index (platelet × neutrophil)/lymphocyte ratio); TC/HDL-C, total-cholesterol-to-high-density-lipoprotein-cholesterol ratio.

**Table 3:** Multivariable logistic regression analysis to explore the relationships between the studied covariates and SOP (vs. control).

SOP Status (326 Affected vs. 136 Controls)			
Independent Covariate	OR (95% CI)	Β (SE)	*p*
Age	6.24 (3.72–10.49)	1.83 (0.26)	3.86 × 10^11^
ECW	7.77 (3.67–16.44)	2.05 (0.38)	0.00000007
Chemerin	2.85 (1.74–4.64)	1.04 (0.25)	0.00002
SII	1.87 (1.23–2.85)	0.62 (0.21)	0.003
TC/HDL-C	3.05 (1.80–5.17)	1.11 (0.26)	0.00002

Data reported as odds ratios with 95% confidence intervals (OR (95% CI)), with corresponding Beta coefficient and standard error (B (SE)). At the initial stage of the study, the following independent variables were tested stepwise forward: age, chemerin, leptin, TC/HDL-C, TG/HDL-C, neutrophils, SII, and ECW. All the variables in the analysis were standardized prior to statistical analysis. Only statistically significant terms are shown. Abbreviations: ECW, extracellular water; SII, systemic immune-inflammation index (platelet × neutrophil)/lymphocyte ratio); SOP, sarcopenic obesity-related phenotype; TC/HDL-C, total-cholesterol-to-high-density-lipoprotein-cholesterol ratio.

**Table 4:** Chi-square and Phi coefficient results for associations between cardiometabolic comorbidities.

Comorbidity Pair	χ^2^	Phi Coefficient	*p*-Value
HTN and HLD	196.703	0.576	<0.001
HLD and T2D	181.254	0.553	<0.001
T2D and HTN	123.988	0.457	<0.001

Abbreviation: χ^2^, chi-square statistics; HLD, hyperlipidemia; HTN, hypertension; T2D, type 2 diabetes.

## Data Availability

Data are contained within the article.

## Supplementary Materials

The following supporting information can be downloaded at: https://www.mdpi.com/article/10.3390/ijms26125699/s1.

## Abbreviations

The following abbreviations are used in this manuscript (in alphabetical order):

**Acronym/** **Abbreviation**	**The Full Term**	**Comment**
BIA	Bioelectrical impedance analysis	Device
BMI	Body mass index	Body composition
DFA	Discriminant function analysis	Statistical analysis method
ECW	Extracellular water	Body composition
FGF-21	Fibroblast growth factor-21	Anti-inflammatory, Growth factor
FM/WT	Fat mass/weight ratio	Body composition
FSTL1	Follistatin-like 1	Pro-inflammatory, Glycoprotein
GDF-15	Growth and differentiation factor-15	Regulatory inflammatory factor
GEE	Generalized estimating equation	Statistical analysis method
HDL-C	High-density lipoprotein cholesterol	Profile lipid
HLD	Hyperlipidemia	Comorbidity
HTN	Hypertension	Comorbidity
MCP1	Monocyte chemoattractant protein 1	Pro-inflammatory, Chemokine
MHR	Monocyte-to-HDL-C ratio	Inflammation index
OB	Obesity	Body composition
SII	Systemic immune-inflammation index	Inflammation index
SIRI	Systemic inflammation response index	Inflammation index
SMM/WT	Skeletal muscle mass/weight ratio	Body composition
SOP	Sarcopenic obesity-related phenotypes	Body composition
SP	Sarcopenia	Body composition
T2D	Type 2 diabetes	Comorbidity
TC	Total cholesterol	Lipid
TC/HDL-C	Total-cholesterol-to-HDL-C ratio	Profile lipid
TG	Triglycerides	Profile lipid
TG/HDL	Triglycerides-to-HDL-C ratio	Profile lipid
WC	Waist circumference	Body composition
